# Brown tumours of a rib initially detected by ultrasound: a case report

**DOI:** 10.1093/bjrcr/uaaf067

**Published:** 2025-12-17

**Authors:** Yuchen Li, Lishan Xiao, Mengmeng Yan, Jiarui Liu, Chunping Ning

**Affiliations:** Department of Abdominal Ultrasound, Affiliated Hospital of Qingdao University, Qingdao 266000, China; Department of Abdominal Ultrasound, Affiliated Hospital of Qingdao University, Qingdao 266000, China; Department of Abdominal Ultrasound, Affiliated Hospital of Qingdao University, Qingdao 266000, China; Department of Abdominal Ultrasound, Affiliated Hospital of Qingdao University, Qingdao 266000, China; Department of Abdominal Ultrasound, Affiliated Hospital of Qingdao University, Qingdao 266000, China

**Keywords:** brown tumour, secondary hyperparathyroidism, rib, ultrasound

## Abstract

Brown tumours, reactive osteolytic lesions caused by hyperparathyroidism, are usually identified by X-ray, computed tomography, and magnetic resonance imaging, with ultrasonography of brown tumours being only rarely reported in the literature. In this paper, we present a case of brown tumours initially detected during ultrasound examination and subsequently confirmed. We describe the ultrasonographic characteristics in detail and discuss the clinical value of ultrasound as a screening and diagnostic tool. Furthermore, we summarise the clinical characteristics, imaging features, and treatment options of brown tumours based on a thorough analysis of the available literature.

## Introduction

Brown tumours,^1^ also known as fibrocystic osteitis, are pseudotumorous lesions of the skeleton formed as a result of reactive fibrous connective tissue hyperplasia caused by primary or secondary hyperparathyroidism. The term “brown tumour” refers to the macroscopically brown appearance, which is caused by an accumulation of hemosiderin pigment. Brown tumour lesions can affect any skeletal structure and may be isolated or exist as multiple foci. They usually affect the craniofacial region, jaw, sternum, pelvis, ribs, or femur.^2^ Most brown tumours are due to primary hyperparathyroidism (more than 80%), but in some patients they are due to secondary hyperparathyroidism caused by chronic renal failure.

Herein, we report a case of brown tumours of the rib in a 34-year-old man with secondary hyperparathyroidism, in which the brown tumours were initially detected during ultrasound examination and then subsequently confirmed.

## Case report

A 34-year-old man presented at our outpatient clinic with a palpable mass in the chest wall. Physical examination revealed a mass of approximately 5.0 × 4.0 cm in the right posterior chest wall with poor mobility and mild tenderness. The mass was painless and growing very slowly.

**Figure 1.**
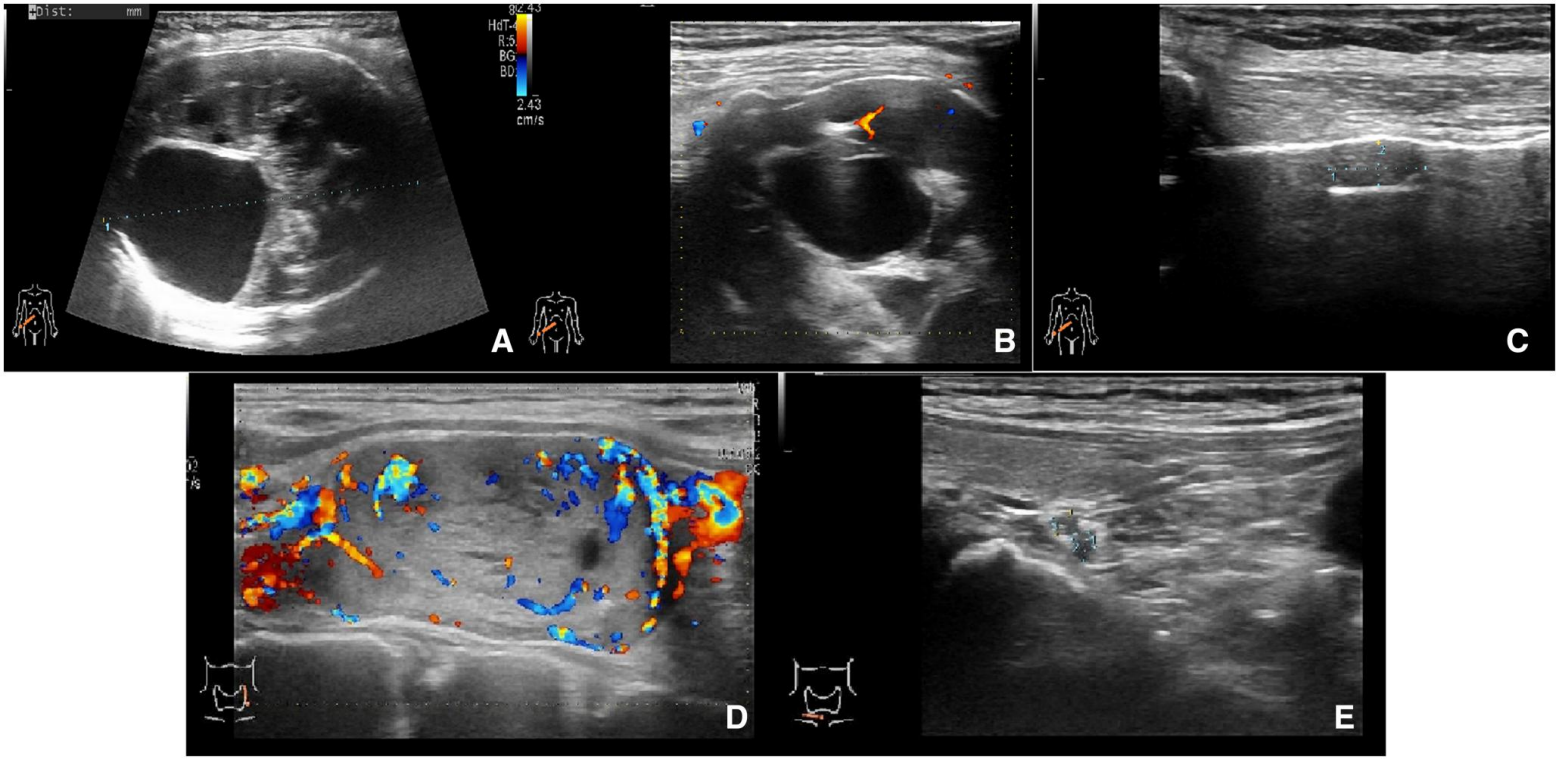
(A-C) Ultrasound shows heterogeneous hypoechoic masses in the tenth rib of the right chest wall, and the colour doppler flow image shows punctate blood flow signal inside the solid part. (D, E) Parathyroid ultrasonography shows heterogeneous/hypoechoic nodules in the left upper and right lower parathyroid glands.

**Figure 2.**
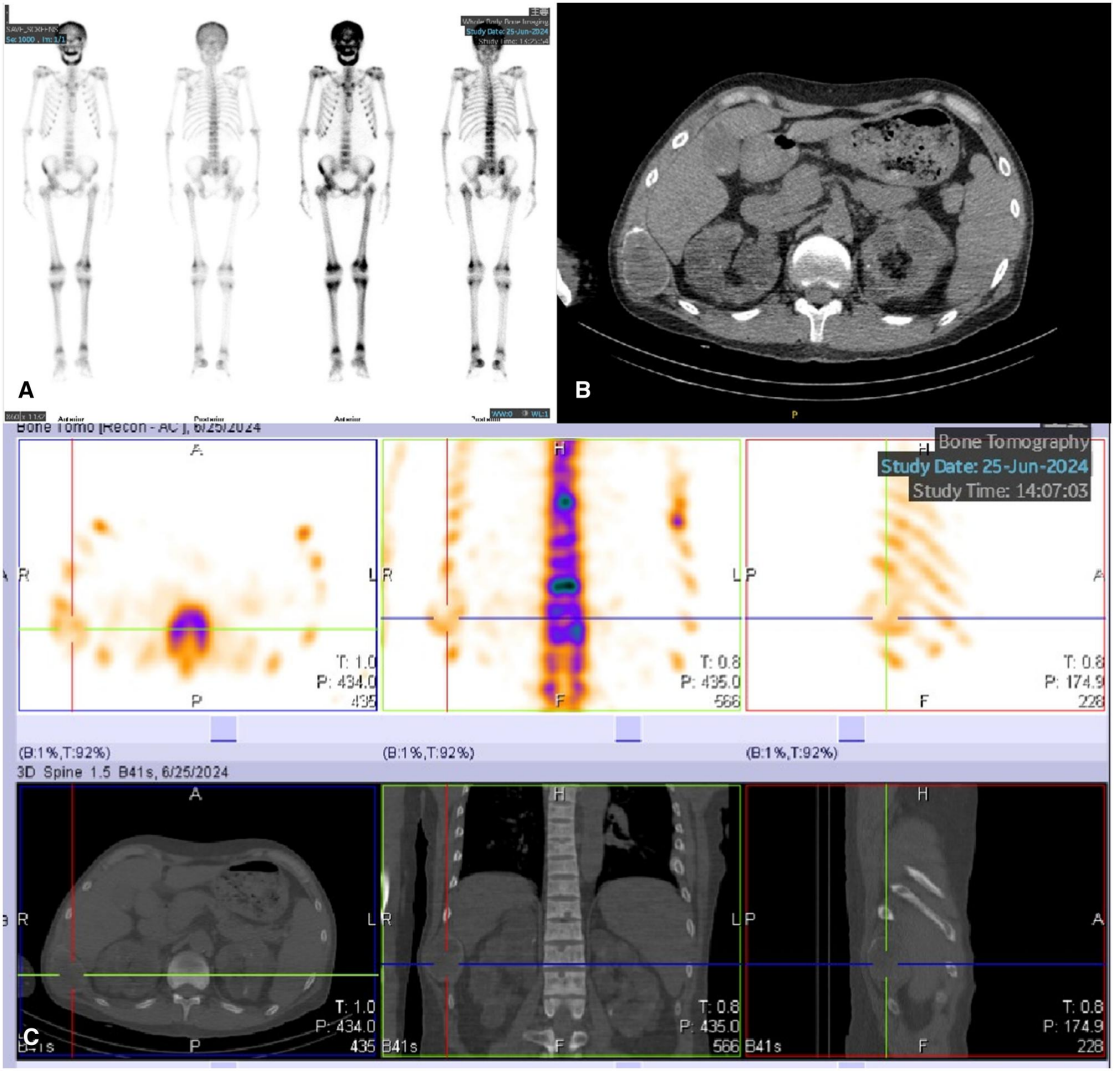
(A) The whole-body bone scan demonstrates diffuse tracer uptake in the skull, with abnormally increased radiotracer accumulation observable in the clavicles, ribs, sternum, scapulae, long bones of the extremities, pelvic bones, and vertebral column. These findings are suggestive of metabolic bone disease, most likely secondary to hyperparathyroid dysfunction. (B, C) SPECT/CT tomographic imaging demonstrates abnormally increased uptake of bone imaging agents. A hyperdense, well-demarcated, expansile osteolytic lesion is observable in the right 10th rib with thinning of the surrounding cortical bone. The left 6th and 7th ribs, as well as the right 11th rib, show irregular morphology with partial expansion, reduced bone density, and evidence of callus formation in some regions. These findings suggest a brown tumour in the right 10th rib and pathological fractures in the left 6th and 7th ribs and right 11th rib.

The patient underwent an ultrasound examination, on which a heterogeneous hypoechoic mass was detected in the right tenth rib, measuring 6.4 × 5.4 × 3.7 cm. The ovoid, heterogeneous, hypoechoic mass had a clear and smooth margin, and multiple cystic changes could be seen inside. Where it had grown expansively, the surface of the bone cortex was thinner but still continuous. No periosteal reaction or noticeable swelling of the surrounding soft tissue was found. Colour Doppler imaging showed punctate blood flow signals inside the mass. Furthermore, another small heterogeneous hypoechoic mass, measuring approximately 0.9 × 0.5 × 0.4 cm, was found adjacent (Figure 1). The patient had been receiving regular haemodialysis treatment for 12 years because of chronic renal insufficiency. Considering the possibility of brown tumours secondary to hyperparathyroidism, we suggested a thyroid ultrasound examination, on which a 2.5 × 2.0 × 3.3 cm heterogeneous nodule was found in the left upper parathyroid region. This nodule was regular with clear borders, and abundant blood flow signals were seen inside it. In addition, two hypoechoic solid nodules were detected in the right lower parathyroid glands, measuring 0.2 × 0.2 cm and 0.2 × 0.3 cm. Therefore, we suspected hyperplasia in the left upper and right lower parathyroid glands and possible brown tumours in the right tenth rib. The patient subsequently underwent chest computed tomography (CT), whole-body bone imaging, and single-photon emission computed tomography (SPECT) (Figure 2). Laboratory tests showed levels of parathyroid hormone of 433.00 pg/mL, blood calcium of 2.21 mmol/L, and blood phosphorus of 1.53 mmol/L. The radiologic and laboratory findings confirmed the initial suspicion of brown tumours and hyperparathyroidism.

## Discussion

Brown tumours are rare, benign tumour-like lesions of the bone and were first described by Henry Jaffe in 1942.^3^ Brown tumours are usually caused by primary or secondary hyperparathyroidism.^4^^,^^5^ Diagnosis of brown tumour depends on clinical manifestations, imaging data, biochemical tests, and pathological examination.

Brown tumour usually presents as an asymptomatic swollen or painful exophytic mass and may be either isolated or multifocal. It may cause pathological fractures or bone pain. Histopathological characteristics of brown tumour include multinucleated osteoclast-type giant cells scattered among mononuclear spindle cells lacking atypia or sarcomatoid features. The brown colour seen in pathohistological analysis is caused by excess osteoclast activity due to massive secretion of parathyroid hormone, intralesional haemorrhage, and hemosiderin deposition.^6^ The histopathology of brown tumour is not specific and is similar to other giant cell lesions such as giant cell tumour of bone, central giant cell reparative granuloma, low-grade osteosarcoma, and primary or secondary aneurysmal bone cysts. Therefore, histology is usually insufficient to confirm the diagnosis.

Imaging is an important tool in the assessment of musculoskeletal disease. Currently, the preferred imaging modality for brown tumours is radiography. On X-ray and CT, brown tumours appear as hyperdense well-demarcated, expansile lytic lesions with variable degrees of bone destruction. The bone cortex may be destroyed and thinned. The tumour is usually highly enhanced on contrast-enhanced CT. The magnetic resonance imaging appearance of brown tumour is often described as isointense or hypointense on T1-weighted images and as hyperintense or hypointense on T2-weighted images.^7^ Multifocal brown tumours can be misdiagnosed as metastatic bone tumour or multiple myeloma on radiological imaging.

The clinical manifestation of bone tumour is usually a superficial mass, and high-frequency ultrasound has gradually become the imaging modality of choice for superficial soft tissue masses. With improvements in the resolution of high-frequency ultrasound and the clinical application of high-frequency wide-field imaging, the value of ultrasound in the evaluation of superficial bone tumours is becoming increasingly clear. Normally, ultrasound cannot be used to visualise bone lesions because it is difficult for it to penetrate the bone cortex. However, brown tumours are osteolytic lesions, and the thinning and destruction of the bone cortex enables ultrasound waves to pass through, making it a feasible method for the clinical evaluation of brown tumours. Ultrasound features of brown tumours have rarely been reported in the literature. Zhang^8^ described the ultrasound features of two patients with brown tumours and suggested the usefulness of ultrasound in the retrospective evaluation of these tumours. The ultrasonographic features of the brown tumour described in this case are similar to those reported by Zhang, but our description is more detailed and comprehensive. Moreover, on ultrasound imaging, brown tumours need be differentiated from other osseous neoplasms. Bone cysts typically manifest as localized areas of osseous expansion, feature anechoic zones within the bone matrix, and present with well-defined margins without any erosion of the adjacent bone cortex. Giant cell tumour of the bone is distinguished by eccentric, expansive, and osteolytic lesions localized at the metaphyseal regions of long bones, with a thinned yet continuous bone cortex. Osteosarcoma is characterised by serpiginous cortical destruction in the metaphyseal regions of long bones, along with the development of irregular hypoechoic soft tissue masses, and is accompanied by a marked periosteal reaction. Metastatic bone tumours are generally associated with a prior history of malignancy, are predominantly solid in consistency, exhibit rapid growth, demonstrate cortical destruction or continuous disruption of the bone structure, and manifest abundant vascularity on colour Doppler imaging. In contrast to other imaging modalities, ultrasound offers several advantages in the initial assessment of suspected brown tumours. First, ultrasound can detect bone swelling, thinning of the bone cortex caused by resorption of the subperiosteal bone, or a mass with destruction of the bone cortex. Second, ultrasound can provide crucial diagnostic clues through examination of the parathyroid region at the same time as the suspected brown tumour lesion. Third, ultrasound can be used to assess the progress of the lesion at any time during treatment because of its convenience, freedom from ionising radiation, and relatively low cost.

The treatment of brown tumours usually focuses on managing the underlying hyperparathyroidism, with different strategies for primary and secondary hyperparathyroidism.^9^ Parathyroidectomy is the gold-standard treatment for brown tumours in patients with primary hyperparathyroidism, while the mainstay of treatment for secondary hyperparathyroidism in patients with dialysis-dependent chronic kidney disease is a combination of phosphate binders, active vitamin D analogs, and calcimimetics, guided by close monitoring of serum calcium, phosphate, and parathyroid hormone levels. However, surgery may be required in refractory cases that do not respond to conservative treatment or in the case of larger symptomatic lesions.^10^ In this case, the patient presented with only a right-sided chest wall mass without other uncomfortable symptoms. He was treated conservatively with close follow-up and observation without surgical intervention.

## Conclusion

Brown tumours are rare non-neoplastic osteolytic bone lesions. Compared with conventional radiography, ultrasonography can serve as a valuable initial imaging tool in the diagnostic workup of brown tumours. In this case report, we highlight the characteristic sonographic features of these lesions and underscore the clinical utility of ultrasound in their identification and differential diagnosis.

## Learning points

- Brown tumour is a rare complication of hyperparathyroidism.
- On sonography, a brown tumour typically presents as a well-circumscribed, oval or round, heterogeneous hypoechoic mass with cystic areas and colour flow signals in its solid components.
- Understanding and recognising the ultrasonographic features of brown tumours can provide valuable assistance in clinical diagnosis.
